# Supplementary material for: Iterative improvement in the automatic modular design of robot swarms
Source: PeerJ Comput Sci. 2020 Dec 7;6:e322. doi: 10.7717/peerj-cs.322 (PMC7924708; doi:10.7717/peerj-cs.322)
Supplement: Supplemental Information 3 [file peerj-cs-06-322-s003.zip › argos3/doc/api/standalone/a00544_source.html]

ARGoS: plugins/robots/generic/simulator/range\_and\_bearing\_medium\_sensor.cpp Source File


- Main Page
- Related Pages
- Namespaces
- Classes
- Files

- File List
- File Members

# plugins/robots/generic/simulator/range\_and\_bearing\_medium\_sensor.cpp

Go to the documentation of this file.

```
00001 
00007 #include "range_and_bearing_medium_sensor.h"
00008 #include <argos3/core/simulator/simulator.h>
00009 #include <argos3/core/simulator/entity/composable_entity.h>
00010 #include <argos3/core/simulator/entity/controllable_entity.h>
00011 #include <argos3/plugins/simulator/entities/rab_equipped_entity.h>
00012 #include <argos3/plugins/simulator/media/rab_medium.h>
00013 
00014 namespace argos {
00015 
00016    /****************************************/
00017    /****************************************/
00018 
00019    CRange<CRadians> INCLINATION_RANGE(CRadians(0), CRadians(ARGOS_PI));
00020 
00021    /****************************************/
00022    /****************************************/
00023 
00024    CRangeAndBearingMediumSensor::CRangeAndBearingMediumSensor() :
00025       m_pcRangeAndBearingEquippedEntity(NULL),
00026       m_fDistanceNoiseStdDev(0.0f),
00027       m_fPacketDropProb(0.0f),
00028       m_pcRNG(NULL),
00029       m_cSpace(CSimulator::GetInstance().GetSpace()),
00030       m_bShowRays(false) {}
00031 
00032    /****************************************/
00033    /****************************************/
00034 
00035    void CRangeAndBearingMediumSensor::SetRobot(CComposableEntity& c_entity) {
00036       /* Assign RAB equipped entity to this sensor */
00037       m_pcRangeAndBearingEquippedEntity = &c_entity.GetComponent<CRABEquippedEntity>("rab");
00038       /* Enable the RAB equipped entity */
00039       m_pcRangeAndBearingEquippedEntity->Enable();
00040       /* Get reference to controllable entity */
00041       m_pcControllableEntity = &c_entity.GetComponent<CControllableEntity>("controller");
00042    }
00043 
00044    /****************************************/
00045    /****************************************/
00046 
00047    void CRangeAndBearingMediumSensor::Init(TConfigurationNode& t_tree) {
00048       try {
00049          /* Parent class init */
00050          CCI_RangeAndBearingSensor::Init(t_tree);
00051          /* Show rays? */
00052          GetNodeAttributeOrDefault(t_tree, "show_rays", m_bShowRays, m_bShowRays);
00053          /* Parse noise */
00054          GetNodeAttributeOrDefault(t_tree, "noise_std_dev", m_fDistanceNoiseStdDev, m_fDistanceNoiseStdDev);
00055          GetNodeAttributeOrDefault(t_tree, "packet_drop_prob", m_fPacketDropProb, m_fPacketDropProb);
00056          if((m_fPacketDropProb > 0.0f) ||
00057             (m_fDistanceNoiseStdDev > 0.0f)) {
00058             m_pcRNG = CRandom::CreateRNG("argos");
00059          }
00060          /* Get RAB medium from id specified in the XML */
00061          std::string strMedium;
00062          GetNodeAttribute(t_tree, "medium", strMedium);
00063          m_pcRangeAndBearingMedium = &(CSimulator::GetInstance().GetMedium<CRABMedium>(strMedium));
00064          /* Assign RAB entity to the medium */
00065          m_pcRangeAndBearingMedium->AddEntity(*m_pcRangeAndBearingEquippedEntity);
00066       }
00067       catch(CARGoSException& ex) {
00068          THROW_ARGOSEXCEPTION_NESTED("Error initializing the range and bearing medium sensor", ex);
00069       }
00070    }
00071 
00072    /****************************************/
00073    /****************************************/
00074 
00075    void CRangeAndBearingMediumSensor::Update() {
00077       /* Delete old readings */
00078       m_tReadings.clear();
00079       /* Get list of communicating RABs */
00080       const CSet<CRABEquippedEntity*>& setRABs = m_pcRangeAndBearingMedium->GetRABsCommunicatingWith(*m_pcRangeAndBearingEquippedEntity);
00081       /* Buffer for calculating the message--robot distance */
00082       CVector3 cVectorRobotToMessage;
00083       /* Buffer for the received packet */
00084       CCI_RangeAndBearingSensor::SPacket sPacket;
00085       /* Go through communicating RABs and create packets */
00086       for(CSet<CRABEquippedEntity*>::iterator it = setRABs.begin();
00087           it != setRABs.end(); ++it) {
00088          /* Should we drop this packet? */
00089          if(m_pcRNG == NULL || /* No noise to apply */
00090             !(m_fPacketDropProb > 0.0f &&
00091               m_pcRNG->Bernoulli(m_fPacketDropProb)) /* Packet is not dropped */
00092             ) {
00093             /* Create a reference to the RAB entity to process */
00094             CRABEquippedEntity& cRABEntity = **it;
00095             /* Add ray if requested */
00096             if(m_bShowRays) {
00097                m_pcControllableEntity->AddCheckedRay(false,
00098                                                      CRay3(cRABEntity.GetPosition(),
00099                                                            m_pcRangeAndBearingEquippedEntity->GetPosition()));
00100             }
00101             /* Calculate vector to entity */
00102             cVectorRobotToMessage = cRABEntity.GetPosition();
00103             cVectorRobotToMessage -= m_pcRangeAndBearingEquippedEntity->GetPosition();
00104             /* If noise was setup, add it */
00105             if(m_pcRNG && m_fDistanceNoiseStdDev > 0.0f) {
00106                cVectorRobotToMessage += CVector3(
00107                   m_pcRNG->Gaussian(m_fDistanceNoiseStdDev),
00108                   m_pcRNG->Uniform(INCLINATION_RANGE),
00109                   m_pcRNG->Uniform(CRadians::UNSIGNED_RANGE));
00110             }
00111             /*
00112              * Set range and bearing from cVectorRobotToMessage
00113              * First, we must rotate the cVectorRobotToMessage so that
00114              * it is local to the robot coordinate system. To do this,
00115              * it enough to rotate cVectorRobotToMessage by the inverse
00116              * of the robot orientation.
00117              */
00118             cVectorRobotToMessage.Rotate(m_pcRangeAndBearingEquippedEntity->GetOrientation().Inverse());
00119             cVectorRobotToMessage.ToSphericalCoords(sPacket.Range,
00120                                                     sPacket.VerticalBearing,
00121                                                     sPacket.HorizontalBearing);
00122             /* Convert range to cm */
00123             sPacket.Range *= 100.0f;
00124             /* Normalize horizontal bearing between [-pi,pi] */
00125             sPacket.HorizontalBearing.SignedNormalize();
00126             /*
00127              * The vertical bearing is defined as the angle between the local
00128              * robot XY plane and the message source position, i.e., the elevation
00129              * in math jargon. However, cVectorRobotToMessage.ToSphericalCoords()
00130              * sets sPacket.VerticalBearing to the inclination, which is the angle
00131              * between the azimuth vector (robot local Z axis) and
00132              * cVectorRobotToMessage. Elevation = 90 degrees - Inclination.
00133              */
00134             sPacket.VerticalBearing.Negate();
00135             sPacket.VerticalBearing += CRadians::PI_OVER_TWO;
00136             sPacket.VerticalBearing.SignedNormalize();
00137             /* Set message data */
00138             sPacket.Data = cRABEntity.GetData();
00139             /* Add message to the list */
00140             m_tReadings.push_back(sPacket);
00141          }
00142       }
00143    }
00144       
00145    /****************************************/
00146    /****************************************/
00147 
00148    void CRangeAndBearingMediumSensor::Reset() {
00149       m_tReadings.clear();
00150    }
00151 
00152    /****************************************/
00153    /****************************************/
00154 
00155    void CRangeAndBearingMediumSensor::Destroy() {
00156       m_pcRangeAndBearingMedium->RemoveEntity(*m_pcRangeAndBearingEquippedEntity);
00157    }
00158 
00159    /****************************************/
00160    /****************************************/
00161 
00162    REGISTER_SENSOR(CRangeAndBearingMediumSensor,
00163                    "range_and_bearing", "medium",
00164                    "Carlo Pinciroli [ilpincy@gmail.com]",
00165                    "1.0",
00166                    "The range-and-bearing sensor.",
00167                    "This sensor allows robots to perform situated communication, i.e., a form of\n"
00168                    "wireless communication whereby the receiver also knows the location of the\n"
00169                    "sender with respect to its own frame of reference.\n"
00170                    "This implementation of the range-and-bearing sensor is associated to the\n"
00171                    "range-and-bearing medium. To be able to use this sensor, you must add a\n"
00172                    "range-and-bearing medium to the <media> section.\n"
00173                    "This sensor allows a robot to receive messages. To send messages, you need the\n"
00174                    "range-and-bearing actuator.\n"
00175                    "To use this sensor, in controllers you must include the\n"
00176                    "ci_range_and_bearing_sensor.h header.\n\n"
00177                    "REQUIRED XML CONFIGURATION\n\n"
00178                    "  <controllers>\n"
00179                    "    ...\n"
00180                    "    <my_controller ...>\n"
00181                    "      ...\n"
00182                    "      <sensors>\n"
00183                    "        ...\n"
00184                    "        <range_and_bearing implementation=\"medium\"\n"
00185                    "                           medium=\"rab\" />\n"
00186                    "        ...\n"
00187                    "      </sensors>\n"
00188                    "      ...\n"
00189                    "    </my_controller>\n"
00190                    "    ...\n"
00191                    "  </controllers>\n\n"
00192                    "The 'medium' attribute must be set to the id of the range-and-bearing medium\n"
00193                    "declared in the <media> section.\n\n"
00194                    "OPTIONAL XML CONFIGURATION\n\n"
00195                    "It is possible to draw the rays shot by the range-and-bearing sensor in the\n"
00196                    "OpenGL visualization. This can be useful for sensor debugging but also to\n"
00197                    "understand what's wrong in your controller. In OpenGL, the rays are drawn in\n"
00198                    "cyan when two robots are communicating.\n"
00199                    "To turn this functionality on, add the attribute \"show_rays\" as in this\n"
00200                    "example:\n\n"
00201                    "  <controllers>\n"
00202                    "    ...\n"
00203                    "    <my_controller ...>\n"
00204                    "      ...\n"
00205                    "      <sensors>\n"
00206                    "        ...\n"
00207                    "        <range_and_bearing implementation=\"medium\"\n"
00208                    "                           medium=\"rab\"\n"
00209                    "                           show_rays=\"true\" />\n"
00210                    "        ...\n"
00211                    "      </sensors>\n"
00212                    "      ...\n"
00213                    "    </my_controller>\n"
00214                    "    ...\n"
00215                    "  </controllers>\n\n"
00216                    "It is possible to add noise to the readings, thus matching the characteristics\n"
00217                    "of a real robot better. Noise is implemented as a random vector added to the\n"
00218                    "vector joining two communicating robots. For the random vector, the inclination\n"
00219                    "and azimuth are chosen uniformly in the range [0:PI] and [0:2PI], respectively,\n"
00220                    "and the length is drawn from a Gaussian distribution. The standard deviation of\n"
00221                    "the Gaussian distribution is expressed in meters and set by the user through\n"
00222                    "the attribute 'noise_std_dev' as shown in this example:\n\n"
00223                    "  <controllers>\n"
00224                    "    ...\n"
00225                    "    <my_controller ...>\n"
00226                    "      ...\n"
00227                    "      <sensors>\n"
00228                    "        ...\n"
00229                    "        <range_and_bearing implementation=\"medium\"\n"
00230                    "                           medium=\"rab\"\n"
00231                    "                           noise_std_dev=\"0.1\" />\n"
00232                    "        ...\n"
00233                    "      </sensors>\n"
00234                    "      ...\n"
00235                    "    </my_controller>\n"
00236                    "    ...\n"
00237                    "  </controllers>\n\n"
00238                    "In addition, it is possible to specify the probability that a packet gets lost\n"
00239                    "even though the robot should have received it (i.e., packet dropping). To set\n"
00240                    "this probability, use the attribute 'packet_drop_prob' as shown in the example:\n"
00241                    "  <controllers>\n"
00242                    "    ...\n"
00243                    "    <my_controller ...>\n"
00244                    "      ...\n"
00245                    "      <sensors>\n"
00246                    "        ...\n"
00247                    "        <range_and_bearing implementation=\"medium\"\n"
00248                    "                           medium=\"rab\"\n"
00249                    "                           packet_drop_prob=\"0.1\" />\n"
00250                    "        ...\n"
00251                    "      </sensors>\n"
00252                    "      ...\n"
00253                    "    </my_controller>\n"
00254                    "    ...\n"
00255                    "  </controllers>\n" ,
00256                    "Usable");
00257    
00258 }
```

---

Generated on 10 Jul 2018 for ARGoS by 
 1.6.1
